# Beyond Rejection: Exploring Tacrolimus's Hidden Potential in Toxoplasmosis

**DOI:** 10.7759/cureus.91721

**Published:** 2025-09-06

**Authors:** Benjamin R Johnson, Anna K Potter

**Affiliations:** 1 Emergency Medicine, Nova Southeastern University Dr. Kiran C. Patel College of Osteopathic Medicine, Clearwater, USA; 2 Foundational Sciences, Nova Southeastern University Dr. Kiran C. Patel College of Osteopathic Medicine, Clearwater, USA

**Keywords:** antiparasitic, calcineurin inhibitors, cyclosporin a, tacrolimus, toxoplasma gondii, toxoplasmosis

## Abstract

Human infection and treatment of *Toxoplasma gondii*, known as toxoplasmosis, pose unique challenges and risks, particularly in transplant recipients and immunocompromised individuals. *Toxoplasma gondii* is an apicomplexan protozoan parasite that has been defined as the most successful parasite, with infections ranging from asymptomatic to fatal. It is prevalent throughout the world, presenting higher morbidity and mortality in immunocompromised and immunosuppressed individuals. Tacrolimus is a calcineurin inhibitor, often serving a role in transplant medicine as an immunosuppressant. Cyclosporine is the other commonly used calcineurin inhibitor, with similarity to tacrolimus in its mechanism of action, and has shown to be effective in the treatment of several parasitic infections. Both of these drugs block the intracellular calcium signaling cascade mediated by calcineurin, through binding to their respective immunophilin proteins. *Toxoplasma gondii* is dependent on this pathway for cell lysis and its egress from the vacuole. Cyclosporine has presented antiparasitic activity both in vitro and in vivo against another protozoan parasite with similarities to *T. gondii*, *Plasmodium falciparum*, through its inhibition of this pathway. Given the functional similarities between cyclosporine and tacrolimus, this narrative review aims to assess the antiparasitic potential of tacrolimus, particularly considering the promising antiparasitic activity observed with cyclosporine and other calcineurin inhibitors. Further research is needed on this role in tacrolimus to evaluate its efficacy, safety, and other potentials in the management of toxoplasmosis, independently and in combination therapies.

## Introduction and background

Toxoplasmosis is a pathogenesis due to infection from the *Toxoplasma gondii* (*T. gondii*) protozoan parasite. This apicomplexan parasite infects up to one-third of the world’s population, with effects ranging from asymptomatic infection to death [1]. It has been classified as one of the most successful parasites due to its ability to infect all endotherms worldwide, with the cat being the definitive host [1]. With such a high pathogenicity, organ transplantation is a unique circumstance that can result in reactivation of latent infection following immunosuppression post-transplantation, particularly through inhibition of the cytosolic calcium signaling pathway utilized by the parasite for egress, or inoculation of *T. gondii* within transplanted tissues, such as the brain, cardiac, skeletal muscle, and retina [2]. Other immunosuppressive conditions, such as human immunodeficiency virus (HIV), as well as pregnancy can increase susceptibility to opportunistic infection [3]. Given the potential severity of toxoplasmosis in these vulnerable populations, identifying effective treatment strategies remains essential for optimal clinical management.

Calcineurin is an essential enzyme in eukaryotes, possessing a catalytic subunit, called calcineurin A, and a regulatory subunit, called calcineurin B. This serine/threonine phosphatase is primarily known, in eukaryotes, to be involved in the regulation of calcium homeostasis. When combined with their respective immunophilin proteins, immunosuppressive medications, such as cyclosporine and FK506 (tacrolimus), are able to inhibit the calcium-dependent eukaryotic signal transduction pathway that is regulated by calcineurin [4]. Many of the apicomplexan parasites, including *T. gondii* and its close relative *Plasmodium falciparum*, have been found to directly utilize the calcium that is released by the calcineurin pathway and regulated by these immunophilins, suggesting their dependence on intracellular calcium to break their vacuole and lyse the cell, allowing for proliferation in the host [5]. Given the inhibition of the calcium release pathway by the aforementioned immunosuppressants, these drugs have been identified as possible targets for the treatment of apicomplexan parasitic infection.

Tacrolimus (Tac), also referred to as FK506, is a potent calcineurin inhibitor used as an immunosuppressant following organ transplantation. It is a first line treatment for transplantations of the kidney, heart, lung, intestines, and bone marrow, and is primarily ingested orally for absorption in the gastrointestinal tract, with subsequent metabolization in the liver [6]. After binding to FK-binding protein (FKBP), the drug-immunophilin complex interacts with and inhibits calcineurin. This inhibitory action blocks the relocation of the nuclear factor of activated T cells (NF-AT), resulting in the inability to activate the genes regulated by the NF-AT transcription factor. This inhibition lowers levels of immune signaling molecules responsible for B and T cell proliferation, such as interleukin-4 (IL-4) and CD40 ligand [7]. While this downregulates the innate immune system, the inhibitory action on calcineurin has shown potential to impede the growth and proliferation of intracellular parasites reliant on the calcium release from calcineurin. Given this inhibitory quality, and *T. gondii*’s reliance on intracellular calcium, tacrolimus may hold therapeutic potential in the treatment of *T. gondii*.

## Review

Toxoplasmosis: pathophysiology and treatment challenges

*Toxoplasma gondii* is one of the most successful parasites known and has a broad host range, thought to be able to infect all endotherms around the world. *Toxoplasma gondii*’s zoonotic properties have also been extensively studied due to its ability to cause pregnancy loss and congenital anomalies in the intermediate host, primarily warm-blooded animals. The definitive host of *T. gondii *is the family Felidae, known for its infection of the domesticated cat. Humans can acquire toxoplasmosis through multiple routes, most commonly via foodborne exposure and through the fecal-oral transmission of oocysts. Vertical transmission from delivering the mother to the fetus is rare and often results in loss of the newborn [8]. As an obligate intracellular parasite, infection of a healthy host often results in the formation of cysts in the infected organ, allowing the bradyzoite form to survive and grow until its egress into the system, often upon immunosuppression. The success of *T. gondii *within the host is directly associated with changes in intracellular calcium, suggested to be linked directly to the calcineurin pathway [9]. Immunocompromised and immunosuppressed individuals are subsequently at high risk for infection due to their impaired calcium-signaling and release pathways.

Current clinical practice suggests treatment of toxoplasmosis in the immunocompetent individual with the combination of two antimicrobials, commonly dihydrofolate reductase inhibitors, such as pyrimethamine and trimethoprim, and dihydropteroate synthetase inhibitors, such as sulfonamides. With approximately 11% of the United States population existing with a dormant, asymptomatic, *T. gondii* infection, the primary concern for treatment surrounds those individuals who are immunocompromised or immunosuppressed [10]. Following acute infection, the actively replicating tachyzoites encyst in tissues, primarily the brain, cardiac muscle, skeletal muscle, and retina, as bradyzoites. These cysts remain for the duration of the intermediate host's life, and there is no documented treatment for the eradication of *T. gondii *in its bradyzoite cyst stage. Available therapies function to treat the actively replicating tachyzoite stage of *T. gondii *infection and have been noted to have several adverse reactions and side effects on the treated host, including leukopenia, thrombocytopenia, cutaneous rash, and fever [10]. Treatment of immunocompromised individuals is often supplemented with additional therapies in an attempt to limit some of these side effects.

Toxoplasmosis during transplantation primarily occurs secondary to serological mismatch, with a seronegative recipient receiving a transplant from a seropositive donor. In these cases, clinical manifestation typically occurs within the three months following transplantation [11]. Research indicates that immunosuppressive therapies may induce a reactivation of the infection within already infected hosts; however, there does not appear to be evidence identifying a direct correlation between immunosuppression with tacrolimus, and subsequent toxoplasmosis, following transplantation with serologically mismatched recipients and donors [12]. This lack of research does not suggest that there is a correlation in these cases; however, it does present an area for further evaluation in the role of immunosuppressant medications and toxoplasmosis during transplantation. Traditional treatment of acute toxoplasmosis in transplant recipients is based on the treatment for disseminated toxoplasmosis in patients with immunodeficiency syndrome (AIDS). These patients are suggested to be treated with an induction therapy of high-dose pyrimethamine, sulfadiazine, and leucovorin for six weeks, with subsequent chronic, lifelong suppressive therapy with lower doses of the same. These patients may also receive prophylactic treatments for pneumocystis using a combination of trimethoprim and sulfamethoxazole [13]. Unfortunately, mutations in parasitic enzymes and proteins have resulted in its resistance to sulfa drugs, given their profoundly similar affinity to human and parasitic enzymes alike. With increasing drug resistance and high doses required for current treatments of these parasites, combined with side effects such as drug-induced arrythmias and nephrotoxicity, new strategies for the management of toxoplasmosis are needed [14]. Thorough consideration should be given to the treatment of these patients due to frequent comorbidities that could be additionally impacted by treatment with currently available therapies.

Tacrolimus: mechanism of action and immunosuppressive properties

Tacrolimus binds to a highly conserved intracellular receptor known as FKBP12. This receptor has been found in practically every eukaryotic cell type and tissue from yeast to human, with nominal changes in amino acid sequencing. An enzymatic property of this protein receptor catalyzes the cis-to-trans isomerization of peptidyl-prolyl bonds. This enzymatic behavior of the receptor suggests that tacrolimus alone is a prodrug, requiring binding with the FKB12 receptor to become active. It is suggested that, upon activation, the FK-506/FKB dimer complex bind to an inhibitory site of calcineurin, impeding the downstream activation of calcineurin and release of calcium. Considering that calcineurin-mediated calcium release is directly related to the transcription of several lymphokine genes, such as interleukin-2 (IL-2), interleukin-3 (IL-3), interleukin-4 (IL-4), tumor necrosis factor-alpha (TNF-alpha), and interferon-gamma (IFN-gamma) [15], it is proposed that tacrolimus has immunosuppressive properties through the downregulation of these genes.

The exact function of T cell regulation is not understood; however, the immunosuppressive function is believed to be dependent on the inhibition of T cell activation. When T cells receive activating signals from the T cell receptor (TCR), intracellular calcium is released, subsequently activating calcineurin to dephosphorylate and activating NFAT. With tacrolimus bound to its respective immunophilin, FKB, the dimer is able to bind to calcineurin, preventing its activation and subsequent dephosphorylation of NFAT. This subsequently results in the downregulation of several key cytokine genes responsible for the survival and proliferation of T cells, including IL-2. Several studies suggest that tacrolimus may also affect already activated CD4+ T cells, possibly by indirectly promoting regulatory T cells through suppressed gene expression [16].

This highly specific mechanism of action, coupled with the presence of similar immunophilins and related proteins in certain parasites, suggests that tacrolimus may have potential as an adjunct treatment in such cases. One study found that the use of tacrolimus in cases of cerebral malaria, caused by the protozoa *Plasmodium falciparum*, can inhibit the infiltration of murine CD8+ T cells, thereby preventing infection in these hosts [17]. Similarly, tacrolimus has also shown promise in the treatment of helminth infections. In vitro and in vivo studies have found that oral administration of tacrolimus effectively inhibits the growth of cysts in mice infected with human cystic echinococcus, with minimal side effects noted [18]. While limited, existing evidence suggests tacrolimus as a promising candidate for managing parasitic infections and calls for deeper investigation.

Tacrolimus and toxoplasmosis: mechanism of potential action

While there were few studies directly evaluating the use of tacrolimus in the management of toxoplasmosis, cyclosporine, another calcineurin inhibitor with a similar immunosuppressive profile, appears to have been extensively studied. Like tacrolimus, cyclosporine also binds to an immunophilin, known as cyclophilin, to inhibit calcineurin activity [7]. Given the similarities in their mechanisms of action, it is plausible that parasites may exhibit a similar response to tacrolimus as they do to cyclosporine.

Several studies have been completed evaluating the efficacy in the treatment of toxoplasmosis with cyclosporine. In vitro studies have shown cyclosporine to inhibit replication and result in the subsequent death of *T. gondii *when found in macrophages; however, despite the directly observable anti-toxoplasma effects of cyclosporine, there appear to be varying results in mortality. This may be attributed to the balance between the therapeutic threshold for its antagonistic activity and its immunosuppressive ability [19]. A similar study comparing the use of traditional antimicrobials, such as clindamycin and sulfa drugs, alone and coupled with cyclosporine found that while cyclosporine did inhibit the growth of *T. gondii* in vitro when found in macrophages, this was only at maximum concentrations able to be achieved in humans. Despite the drugs underlying immunosuppressive nature and relatively high doses required for inhibition of growth, it maintains some antimicrobial properties, suggesting an alternative drug or derivative therein may be worth evaluating [20]. The demonstrated efficacy of cyclosporine in inhibiting *T. gondii *replication in both in vitro and in vivo models highlights the rationale for further investigation into the potential antiparasitic effects of tacrolimus.

Clinical studies and evidence

Current studies may suggest that toxoplasmosis remains suppressed in patients immunosuppressed with tacrolimus alone; however, it can emerge upon the discontinuation of tacrolimus or addition of other immunosuppressive agents. One in vivo study, utilizing a murine model to assess the risk of dissemination or reactivation of infection following immunosuppression with several different drugs, including cyclosporine, found that immunosuppressants did affect the natural infection pathway; however, it did not affect parasitic load nor increase the probability of dissemination of the infection [21]. This was a pertinent finding, given the similarities between cyclosporine and tacrolimus. Several case reports and studies have explored tacrolimus’ association with toxoplasmosis, particularly in the context of reactivation or primary infection following immunosuppression. Donor-recipient mismatch for serum toxoplasmosis can be a fatal finding in patients without a noted history of *T. gondii* infection receiving organs from a positive donor. In one study, a 45-year-old female who was five weeks post-heart transplant and immunosuppressed with tacrolimus presented initially with atypical pneumonia; however, she was found to have negative blood cultures and serology tests. The patient subsequently died three days following admission, and post-mortem evaluation found disseminated toxoplasmosis due to donor-recipient mismatch for toxoplasma serology. Despite this finding, the recipient was not noted to have seroconversion and had two biopsies that were negative for *T. gondii* [22].

Several studies on toxoplasmosis in transplant recipient patients have suggested seroconversion due to donor-recipient mismatch as a primary cause for toxoplasmosis. A cross-sectional analysis of heart transplant recipients diagnosed with toxoplasmosis post-transplantation further enhanced this by showing that 50% of the donors in this study were seropositive for *T. gondii*. Half of the seronegative recipients receiving hearts from donors that were seropositive developed clinical manifestation of toxoplasmosis. All of these patients were treated with the calcineurin inhibitor cyclosporine; however, the study did conclude that the use of this immunosuppressant did not affect the detection of seroconversion [23]. These results emphasize the importance of pre-transplant serological screening for *T. gondii *and, when feasible, the consideration of donor-recipient matching based on serostatus to minimize the risk of transmission.

A more recent study evaluated delayed diagnosis of toxoplasmosis in transplant recipients, 10 of whom were recipients of hematopoietic stem cells and 10 of whom were recipients of solid organs. This study identified a statistically significant delay in diagnosis of the toxoplasmosis in recipients of solid organs when compared to those who received the hematopoietic stem cells. While more than half of these patients died within 90 days of their toxoplasmosis diagnosis, only one of these patients, within the hematopoietic stem cell group, was identified as being immunosuppressed with tacrolimus [24]. This study did not correlate mortality by specific immunosuppressive drugs, and thus it was not possible to identify if the patient receiving tacrolimus died. Similarly, a study identifying a case of neurotoxoplasmosis following kidney transplantation, between a serologically positive matched donor and recipient, identified tacrolimus as the patient’s initial immunosuppressant. This patient was subsequently removed from tacrolimus due to ongoing nephrotoxicity and gastrointestinal symptoms, secondary to an additional immunosuppressant, mycophenolate. They were placed on an alternative immunosuppressive medication called belatacept. Approximately eight months following this transition, the patient was diagnosed with neurotoxoplasmosis and initiated on traditional treatment [25]. Given this patient’s lack of serological presentation while maintained on tacrolimus, followed by a subsequent appearance of infection following their removal, there is justification for further evaluation of tacrolimus’s role in the reactivation of toxoplasmosis. Together, these studies further highlight the persistent gap in literature and research regarding the impact of tacrolimus as well as other immunosuppressants, specifically regarding their role in toxoplasmosis and their associated morbidity and mortality.

Table 1 summarizes several of the aforementioned studies.

**Table 1:** Summary of recent studies and case reports involving tacrolimus and Toxoplasma gondii infections.

Author, Year	Type of Study	Key Findings	Conclusion
Adekunle et al., 2021 [24]	Retrospective cohort study	55% 90-day mortality from diagnosis of toxoplasmosis following transplantation.	Further screening for *T. gondii *is needed for noncardiac transplant recipients.
Li et al., 2021 [26]	In vivo	Post-infection inflammatory cytokines and chemokines were significantly higher than those in serum or cerebrospinal fluid.	Further investigation of immune modulators may be helpful in the diagnosis of ocular toxoplasmosis and identifies potential therapeutic targets.
Mastrobuoni et al., 2012 [22]	Case report	Death from disseminated toxoplasmosis due to donor-positive/recipient-negative serological mismatch. No seroconversion postmortem	Prophylaxictic treatment is recommended in cases of serological mismatch during transplantation. Toxoplasmosis should be considered following heart transplantation with neurological and respiratory symptoms.
Sluiters et al., 1989 [23]	Observational study	50% of seronegative recipients who received a transplant from a seropositive donor developed toxoplasmosis. Of them, 25% died.	Multi-assay of serology (IgG and IgM) improves detection of previous and current infections of *T. gondii.*
Sumyuen et al., 1996 [21]	In vivo	23% of seropositive recipients had serological evidence of infection, however, none developed clinical disease.	Inconclusive results connecting tacrolimus to T. gondii infection.
Vigilante et al., 2025 [25]	Case report	Death from neurotoxoplasmosis following kidney transplantation.	Further research on immune response for opportunistic pathogens in kidney transplant recipients is needed.

Benefits and limitations of tacrolimus for toxoplasmosis

Tacrolimus is widely used as an immunosuppressant in transplant medicine, yet its potential as an antiparasitic agent remains underexplored. As post-transplant toxoplasmosis becomes more common, with limited and complex treatment strategies for the immunosuppressed, there is a growing interest in its potential for alternative uses. Cyclosporine, another calcineurin inhibitor, has already demonstrated antiparasitic effects by binding *T. gondii*-specific immunophilins. These targets share sequence homology to the human counterparts, including those affected by tacrolimus, suggesting potential for more selective drug development. Cyclosporine has also been shown to be effective against *T. gondii*, in both in vitro and in vivo models [20,27]. Based on these findings, it is proposed that future derivatives could preserve antiparasitic action while reducing systemic immunosuppression. For transplant recipients, this may reduce the need for multiple medications as well as prevent infection from donor-recipient serological mismatch.

Tacrolimus does, however, present limitations that complicate its potential use. Most notably, there is a lack of research assessing its efficacy against parasitic infections, unlike cyclosporin A, which has been more extensively studied. At the time of this review, data on tacrolimus come from case reports and small observational studies, emphasizing the need for continued research to better understand both its safety profile and therapeutic potential in this context. Additionally, despite its approved applications, tacrolimus presents known risks, even within the therapeutic range. Cases of nephrotoxicity have led to irreversible renal damage and even graft failure in transplant recipients, while neurotoxic effects include tremors and seizures [28]. Pharmacologically, there are additional challenges, particularly with its shared route of metabolism. The cytochrome P450 3A4 (CYP3A4) enzyme system is primarily utilized, which also processes common medications such as antifungals and macrolide antibiotics. In combination, these drugs can elevate tacrolimus levels, increasing the risk of toxicity, or conversely enhance metabolization and lower plasma concentrations. As a result, combining tacrolimus with other therapies often requires careful monitoring and may limit its feasibility in multi-drug regimens [29,30]. Beyond these pharmacological risks, experimental antiparasitic treatments in already immunocompromised individuals pose significant clinical and ethical challenges. Nonetheless, these factors do present areas of improvement, caution, and potential benefits for the development of tacrolimus derivatives.

Future directions

Despite the promising suggestions from existing studies, significant opportunities remain to expand the current understanding of tacrolimus in the management of toxoplasmosis. While tacrolimus is traditionally used as an immunosuppressant medication in transplant medicine, its molecular similarity to cyclosporine and evidence of anti-parasitic properties against other protozoans support investigation into its possible role in combination, and non-combination, therapies for toxoplasmosis. Bao et al. demonstrated the efficacy of tacrolimus in reducing the severity of cerebral malaria, suggesting that similar antiparasitic mechanisms could be explored with the *T. gondii* model [17]. Moreover, cyclosporine has shown antiparasitic activity against *T. gondii*, reinforcing the rationale for calcineurin-inhibitor based regimens, which present potential to enhance efficacy or reduce toxicity, when compared to traditional agents [27].

Second, ongoing concerns regarding the nephrotoxicity and neurotoxicity of tacrolimus [28,30] necessitate investigation of novel formulations or delivery strategies, particularly in those patients requiring long-term immunosuppression, such as in the case of transplantation. There may be potential in evaluating alternative metabolism pathways for tacrolimus derivatives to allow for more specific dosing and the minimization of drug-induced toxicity [29]. Additionally, there is a need for high-quality studies in order to evaluate the efficacy, safety profile, and therapeutic value of tacrolimus in the treatment, and possibly prevention, of toxoplasmosis. Clinical-based evidence is ongoing and appears to be expanding; however, there remains no standardized protocols. Studies such as those published by Dunay et al. [10] and Mack and McLeod [20] traditionally guided clinical decision making; however, these data do not yet extend to the context of tacrolimus.

Given its immunomodulatory properties and established role in transplant medicine, tacrolimus may hold particular value in the prevention of *T. gondii *reactivation in seropositive, immunocompromised patients. Seroconversion due to donor-recipient mismatch remains a critical concern in solid organ transplantation, with risk of seroconversion significantly higher when compared to matched seropositive donors [31]. Additionally, initial observations have proposed that immunophilin-binding agents, such as calcineurin inhibitors, often used for immunosuppression, may influence latent infection [5]. Future research would benefit from the assessment of tacrolimus’ role in latent infection and whether modifications could balance immunosuppression with enhanced control of infected, but dormant, hosts. These findings support the biological basis and clinical rationale for further investigation of tacrolimus in the management of parasitic infection in both transplant and non-transplant populations.

This hypothesis could be evaluated using a combination of in vitro, in vivo, and clinical studies. Future evaluation should begin with foundational research, most importantly using an in vivo model. In vivo murine models for ocular toxoplasmosis have been used to observe the natural route of infection for this parasite [26]. Similarly, in vitro growth of bradyzoite *T. gondii* in human myotubes and tachyzoites in human fibroblasts has been recently utilized for the measurement of parasitic replication and egress [32]. Models like these have the potential to be treated with tacrolimus and observe subsequent effects such as parasitic burden, survival, and seroconversion. Retrospective cohort studies are feasible to evaluate a correlation between immunosuppressant profiles and toxoplasmosis. Together, these approaches would allow for the simultaneous evaluation of pharmacodynamic effects as well as clinical relevance in transplant recipients treated with tacrolimus.

## Conclusions

Tacrolimus is widely accepted in transplant medicine for its immunosuppressive properties; however, research has begun to explore potential benefits beyond its approved uses. Studies evaluating tacrolimus’s antiparasitic effects against *T. gondii* remain limited, despite existing data, clinical-based observations, and similarities with cyclosporine suggesting that tacrolimus may also have effects on parasitic infections. These early findings suggest that there may be benefit in the further evaluation of tacrolimus in the management of toxoplasmosis. The challenges in treating toxoplasmosis, particularly in immunocompromised individuals, coupled with the rising incidence of post-transplant infections, support further evaluation of the potential of tacrolimus. Research of its antiparasitic activity, improvements to minimize toxicity, and the development of defined clinical standards for study will be crucial to effectively pursue its evaluation. Tacrolimus warrants further investigation as an antiparasitic agent in the treatment of toxoplasmosis.
